# Mind-Reading Ability and Structural Connectivity Changes in Aging

**DOI:** 10.3389/fpsyg.2015.01808

**Published:** 2015-11-26

**Authors:** Monia Cabinio, Federica Rossetto, Valeria Blasi, Federica Savazzi, Ilaria Castelli, Davide Massaro, Annalisa Valle, Raffaello Nemni, Mario Clerici, Antonella Marchetti, Francesca Baglio

**Affiliations:** ^1^IRCCS Fondazione don Carlo Gnocchi ONLUSMilan, Italy; ^2^Research Unit on Theory of Mind, Department of Psychology, Università Cattolica del Sacro CuoreMilan, Italy; ^3^Dipartimento di Scienze Umane e Sociali, Università degli Studi di BergamoBergamo, Italy; ^4^Università degli Studi di MilanoMilan, Italy

**Keywords:** magnetic resonance imaging (MRI), diffusion magnetic resonance imaging, aging neuroscience, growth and development, theory of mind (ToM), voxel-based morphometry (VBM), tract-based spatial statistics, mind-reading

## Abstract

The Mind-Reading ability through the eyes is an important component of the affective Theory of Mind (ToM), which allows people to infer the other’s mental state from the eye gaze. The aim of the present study was to investigate to which extent age-associated structural brain changes impact this ability and to determine if this association is related to executive functions in elderly subjects. For this purpose, Magnetic Resonance Imaging was used to determine both gray matter and white matter (WM) areas associated with aging. The resulting areas have been included in a subsequent correlation analysis to detect the brain regions whose structure was associated with the Mind-Reading ability through the eyes, assessed with the Italian version of the “Reading the Mind in the Eyes” (RME) test, in a sample of 36 healthy subjects ranging from 24 to 79 years of age. The analysis resulted in three important findings: (1) the performance to the RME test is relatively stable across the decades 20–70 (despite a slight decrease of this ability with aging) and independent from executive functions; (2) structural brain imaging demonstrated the involvement of a great number of cortical ToM areas for the execution of the RME test: the bilateral precentral gyrus, the bilateral posterior insula, the left superior temporal gyrus and the left inferior frontal gyrus, which also showed a significant volume decrease with age; (3) an age and task-related decline in WM connectivity on left fronto-temporal portion of the brain. Our results confirm the age-related structural modifications of the brain and show that these changes have an influence on the Mind-Reading ability through the eyes.

## Introduction

Theory of mind (ToM) is the ability to attribute mental states to oneself and others and to understand that others have intentions, desires, and beliefs that are different from one’s own (Premack and Woodruff, 1978). This ability is considered to be essential for social interactions, since it enables individuals to understand and predict the behavior of others, even in the presence of minimal cues, such as a facial expression or the eye gaze (Baron-Cohen et al., 1997a).

The ability to “Mind-Read through the eyes” (Baron-Cohen et al., 1997b) is a widely investigated competence that can be behaviorally assessed using the “Reading the Mind in the Eyes” test (RME test, Baron-Cohen et al., 2001). The RME test requires the attribution of mental states to others via observation of pictures representing only the eye region of the face. It is one of the most used tasks to investigate *affective* ToM, i.e., the ability to understand affective states, emotions and feelings of other people, which is distinguished from *cognitive* ToM, defined as the ability to understand the beliefs, goals, and intentions of others (Shamay-Tsoory et al., 2010; Duval et al., 2011).

The importance of eye gaze in social interaction is noteworthy and the presence of simple abilities such as gaze following (Farroni et al., 2004) and face recognition (McKone et al., 2012; Streri et al., 2013) in newborns shows that humans are hardwired to it. Moreover, there is strong evidence that underlines how the precursors of the Mind-Reading ability through the eyes are present even in the earliest phases of development. In fact, within the first 2 years of life a succession of precursors, i.e., early cognitive structures linked to the understanding of the mind through the eyes, paves the way to the mentalistic competence: the understanding of visual perception (Flavell, 1981, 1988; Wimmer et al., 1988; Gopnik et al., 1994), social referencing (Klinnert, 1984; Sorce et al., 1985), joint attention and pointing (Butterworth, 1991, 1994; Baron-Cohen and Ring, 1994) that combined together into triadic interactions led to declarative pointing (Camaioni, 1993). Specifically, declarative pointing is a key precursor not only of referential language, but also of mentalizing in the preverbal phase of development. Specifically, it demonstrates an early understanding of others having their own mind which can be influenced with non-verbal ways of communication. Taken altogether, these precursors show the importance of the ability to extract information through eye contact for the subsequent development of more and more complex Mind-Reading abilities. In this context, examples include the ability of engaging in recursive thinking, such as first order false belief reasoning around four years of age and second order false belief reasoning around 8–10 years (Baron-Cohen et al., 2001). Even if ToM undergoes the most significant changes during childhood, it would be misleading to consider it as an immutable ability. Rather, changes continue during adolescence and early adulthood (Blakemore et al., 2007; Moor et al., 2012) and in the later years of life (see Sandoz et al., 2014 for a review).

More specifically, the decreased performance in ToM ability in the elderly seems to happen regardless of the type of task employed (eyes, videos, stories, false belief task), of the modality (verbal/visual static, visual dynamic) and of the affective/cognitive content of the task (Henry et al., 2013). The study of the developmental changes of ToM necessarily implies the analyses of the contribution of cognitive processes to the ToM performance. The main cognitive processes considered in relation to ToM changes in the elderly are executive functions, vocabulary, logical reasoning, episodic memory, and speed of processing (Sandoz et al., 2014). The importance of the cognitive decline for ToM abilities is still an open matter of debate. For example, the review by Sandoz et al. (2014) reports a pattern of significant correlations between ToM abilities and cognitive processes depending of the specific type of ToM task employed. However, the connections of ToM performances with executive functioning appear to be the most relevant. This relevance has been also demonstrated at the neural level: Mahy et al. (2014) discuss the “executive function approach” among the main theoretical views on ToM development. There are two main positions concerning the contribution of executive function to ToM reasoning: the first one considers executive function necessary to ToM functioning, while the second one necessary but not sufficient. Furthermore, the two forementioned stances make different predictions about the neural activations during ToM tasks. The review by Moran (2013) shows on the one side a strong interconnections between ToM abilities and cognitive processes in the standard ToM tasks, and on the other side, a relative independence of ToM skills from cognitive processes when the former are assessed through continuous, rather than standard categorical, tasks.

In recent years, neuroimaging techniques have become one of the most powerful tools for studying *in vivo* brain structure and functioning, also in life-span perspective. Many studies have demonstrated that aging is associated with a significant, non-linear decline in gray matter (GM) density (Sowell et al., 2003) and changes in white matter (WM) architecture that can be driven by myelin degeneration (for a review, see Raz et al., 1998). Age-related WM decline impairs cognitive performance (Raz, 2005; Davis et al., 2009). Zuo et al. (2010) demonstrated that functional connectivity decreases with age in homologs areas involved with higher order processing. Moreover, neuroimaging studies have investigated how specific behaviors and abilities, such as ToM, depend on brain structure and physiology. In this view, with the aid of magnetic resonance imaging (MRI), it was possible to investigate brain pathways associated with ToM through the life span. A recent meta-analysis of studies performed with functional MRI (fMRI; Schurz et al., 2014), showed a core cortical network composed of the middle prefrontal cortex and the bilateral temporo-parietal junction (TPJ) active when reasoning about mental states, irrespective of the task- and stimulus-formats. When considering the RME test, the core network involved is predominantly left-sided and includes: inferior frontal gyrus [Brodmann area (BA) 45], medial prefrontal cortex, precentral and middle frontal gyri (BA 6), insula and posterior temporal cortixes (TPJ; Schurz et al., 2014). Beyond these core areas, surrounding regions appear to be involved in specific ToM tasks, such as the precuneus, temporal regions and inferior frontal cortices (see Schurz et al., 2014). This complex circuitry seems to vary in its degree of activity with age, probably due to age-related compensatory mechanisms needed to maintain adequate levels of performance (Castelli et al., 2010). More precisely, in the study by Castelli et al. (2010) both younger and older adults reported comparably good behavioral performances in the RME, showing the maintenance of adequate mindreading abilities with advancing age. However, on the neural level, a relevant change in the activations emerged, with older participants showing a stronger involvement of the linguistic components of the mirror neuron system (MNS) as well as a more bilateral activation of frontal areas compared to the younger group. Regarding brain structure, only one pioneering study (Charlton et al., 2009) directly investigated the correlation between structural brain features and ToM competences using a life span perspective in subjects aged 50–90 years. A correlation was found between WM microstructure (at the whole-brain level) and cognitive ToM abilities shading some light on age-related changes. However, this study focused only on the older age without considering the progressive changes occurring in the earlier phases of the adult age (20–50 years of age).

The main aim of the present study was to investigate how age-related structural brain changes impact affective ToM ability as measured with the RME task. To achieve this goal we used structural MRI techniques aimed at exploring structural connectivity in both GM and WM in a cohort of healthy subjects between 24 and 79 years of age. We expected to find age-related cortical and subcortical modifications. Within these areas, we wanted to assess the presence of task-related structural features that might be associated with age-related modifications in the RME performance. Secondly, we aimed at exploring the possible connections between the performances to the RME task and executive functioning, i.e., the specific cognitive dimension most constantly linked to ToM performances.

## Materials and Methods

### Subjects and Psychological Assessment

**FIGURE 1:**
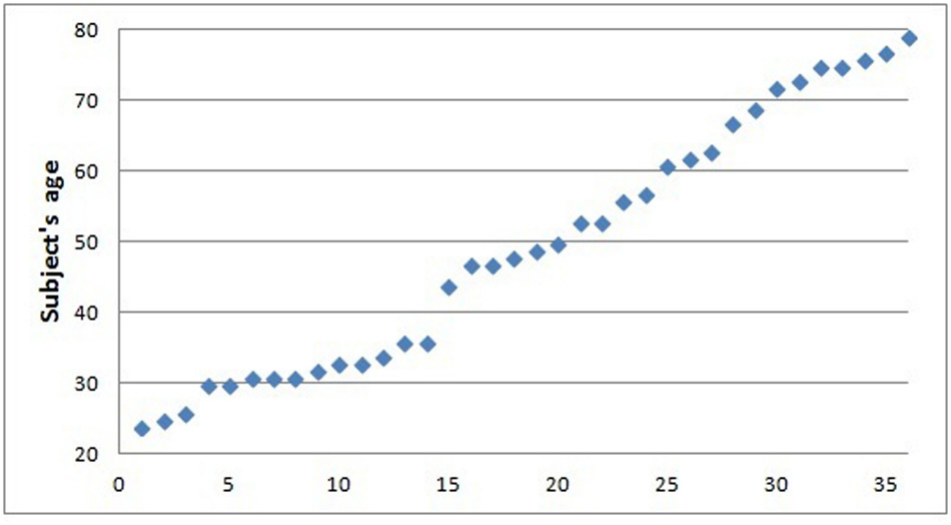
**Scatterplot representing age distribution**.

Thirty-six healthy individuals (13 males, mean age 49.6 ± 17.8, age range 24–79, **Figure 1**) were included in the study. All subjects underwent clinical interview and Mini Mental State Examination test (MMSE; Folstein et al., 1975) to exclude major neurological and/or psychiatric disorders.

All subjects were asked to complete the RME test in its Italian version and the Gender test as a control condition (Serafin and Surian, 2004). The RME test consists of 36 pictures of the eye region from various human faces. Participants have to perform two tasks: the RME proper that consists in choosing what the depicted character is feeling or thinking among four mental states written underneath each picture (cut-off = 13/36, see Baron-Cohen et al., 2001); and the Gender test, in which the same 36 items are presented and the participants are required to indicate the gender. The Gender test is used as a control condition to test basic visual faces discrimination capacities.

In order to test frontal lobe executive functioning, a Letter Fluency Task (LFT; Carlesimo et al., 1996) was performed in a subgroup of elderly subjects (*n* = 13, 3M, mean age 70 ± 7.2, age range: 56–79 years). All the scores obtained from LFT test were corrected for age and the level of education (conversion formulae are reported in Carlesimo et al., 1996).

All subjects also performed a single MRI examination.

### Ethics Statement

The study was approved by the Ethics Committee of Don Gnocchi Foundation, and informed written consent was obtained from all the included subjects before study initiation. The study was conducted within Don Carlo Gnocchi Foundation, IRCCS, Milan (Italy).

### MRI Acquisition

The MRI examination was performed on a 1.5T Siemens scanner. The following sequences were collected: (1) a conventional dual-echo turbo spin echo and fluid-attenuated inversion recovery (FLAIR) sequence (TR/TE = 2920/22 ms, FoV = 240 mm × 180 mm, in-plane resolution = 0.75 mm × 0.75 mm, slice thickness = 4 mm, number of axial slices = 25) and FLAIR sequence (TR/TE = 9000/121 ms, FoV = 240 mm × 168 mm, in-plane resolution = 0.94 mm × 0.94 mm, slice thickness = 5 mm, number of coronal slices = 24), to exclude brain abnormalities; (2) diffusion weighted (DW) single shot spin-echo (TR/TE = 7100/94 ms, 50 axial slices, 128 × 96 matrix, FOV = 320 mm × 240 mm, slice thickness = 2.5 mm), with diffusion gradients (*b*-value = 900 s/mm^2^) applied in 12 non-collinear directions; (3) 3D T1-weighted magnetization prepared rapid gradient echo (TR = 1,900 ms; TE = 3.37 ms; TI = 1,100 ms; flip angle = 15°; 176 contiguous, 1 mm thick axial slices; matrix size = 192 × 256; FOV = 192 mm × 256 mm).

### MRI Data Processing

#### Gray Matter Analyses – Voxel Based Morphometry (VBM)

Structural GM data were analyzed with FSL-VBM (Douaud et al., 2007), an optimized voxel based morphometry (VBM) protocol (Good et al., 2001) carried out with FSL tools (Smith et al., 2004). First, structural images were bias corrected, brain-extracted and GM-segmented before being registered to the MNI 152 standard space using non-linear registration (Andersson et al., 2007a,b). The resulting images were averaged and flipped along the *x*-axis to create a left–right symmetric, study-specific GM template. Second, all native GM images were non-linearly registered to this study-specific template and “modulated” to correct for local expansion (or contraction) due to the non-linear component of the spatial transformation. The modulated GM images were then smoothed with an isotropic Gaussian kernel with a sigma of 3 mm. Brain tissue volume, normalized for subject head size, was estimated with SIENAX (Smith, 2002), part of FSL (Smith et al., 2004). Finally, a voxelwise general linear model (GLM) was applied using permutation-based non-parametric testing, correcting for multiple comparisons across space.

A regression analysis was also conducted at the group level on GM maps to investigate the relationship between brain structure and age. Gender and brain tissue volume were included as covariates of no interest. Results of the statistical analysis, performed using threshold-free cluster enhancement (TFCE) method, were considered as statistically significant if surviving *p* < 0.005_corrected_ threshold and cluster extent of 30 contiguous voxels.

A further analysis was then performed at group level on the cortical areas correlated with age (thresholded at *p* < 0.05_corrected_ level) to investigate possible correlation between cortical volume and RME test. The statistical analysis was performed using the TFCE method, gender and brain tissue volume were included as covariates of no interest. Results were considered statistically significant if surviving *p* < 0.05_corrected_ threshold level and cluster extent of 30 contiguous voxels.

Considering the results of the correlation analysis, we selected all GM regions that survived the *p* < 0.05 _corrected_ threshold and we calculated the number of GM voxels falling within each resulting area in each subject using a specific function (named FSL maths) of the MRI specific software FSL (http://fsl.fmrib.ox.ac.uk/fsl/) and we termed this measure Individual cluster index (ICI). Statistical analyses were then performed, using a dedicated statistical software, to investigate the presence of correlation between ICI and the score of RME test in all subjects. In a subgroup of subject (*n* = 13, 3M, mean age 70 ± 7.2, age range: 56–79 years) the same statistical analysis was performed to test the correlation between ICI and the results of LFT. Results were considered statistically significant if surviving a *p* < 0.05_corrected_ (corresponding to an uncorrected threshold of *p* < 0.008).

#### White Matter Analysis – Tract-Based Spatial Statistics (TBSS)

Voxelwise statistical analysis of the fractional anisotropy (FA) data, an index of microstructural integrity, was carried out using Tract-Based Spatial Statistics (TBSS; Smith et al., 2006), part of FSL (Smith et al., 2004). First, images were corrected for eddy current distortion, then FA images were created by fitting a tensor model to the raw diffusion data, and then brain-extracted using the Brain Extraction Tool (BET; Smith, 2002). All subjects’ FA data were then aligned into a common space by creating a study-specific template using non-linear registration (Andersson et al., 2007a,b), which uses a b-spline representation of the registration warp field (Rueckert et al., 1999). Next, the mean FA image was created and thinned to create a mean FA skeleton which represents the centers of all tracts common to the group. Each subject’s aligned FA data were then projected onto this skeleton and the resulting data fed into voxelwise cross-subject statistics.

A regression analysis was conducted on the skeletonized WM to investigate the relationship between FA values and age. Gender was included as covariate of no interest. Results of the statistical analyses, performed using TFCE method, have been considered as statistically significant if surviving *p* < 0.005_corrected_ threshold.

A further regression analysis has then be performed on the cortical areas correlating with age (thresholded at *p* < 0.05_corrected_ level) to investigate possible correlation with the RME test. The statistical analysis was performed using the TFCE method. Results have been considered statistically significant if surviving *p* < 0.05_corrected_ threshold level.

## Results

### Psychological Assessment

All subjects presented with MMSE score within normal range (MMSE > 28/30) as well as RME test (mean 26 ± 3.89, cut-off 13/36) and RME “gender” test (mean 34.33 ± 1.82, cut-off 19/36).

Results of the LFT in the elderly subgroup were: 34.7 ± 10.7 (mean corrected score).

Statistical analyses showed an inverse correlation between RME score and age (*r* = –0.355, *p* = 0.034), whereas the score at the Gender test did not survive statistical threshold (*r* = –0.326, ns).

Correlation between RME and LFT in the elderly subgroup was not significant (*r* = –0,001, *p* = 0,997).

### Gray Matter Analyses – VBM

Voxel-wise analysis on GM data (VBM) revealed a left-sided inverse correlation between GM cortical volume and age in the precentral gyrus (BA 6), the inferior frontal gyrus (BA 9) and the superior temporal gyrus/insula. An inverse correlation was also found in a small area over the right central sulcus, involving pre- and post-central gyri (**Table 1**). No direct correlation was found between cortical volume and age.

**Table 1:** Voxel-based morphometry (VBM) clusters of inverse correlation between gray matter volume and age (*p* < 0.005_corrected_ threshold).

Cluster size	*P*_max_	Peak cluster MNI coordinates (*x, y, z*)			Center of gravity MNI coordinates (*x, y, z*)			Brain area
308	<0.0001	-52	-12	32	-55.4	-7.58	35.6	Left precentral gyrus
175	0.0001	-46	6	32	-47.6	11.3	32.7	Left inferior frontal gyrus
131	0.0001	-40	-8	-14	-42.5	-7.56	-12	Left superior temporal gyrus
46	0.0001	58	-12	32	56.2	-11.5	36	Right pre/ post central gyrus


**FIGURE 2:**
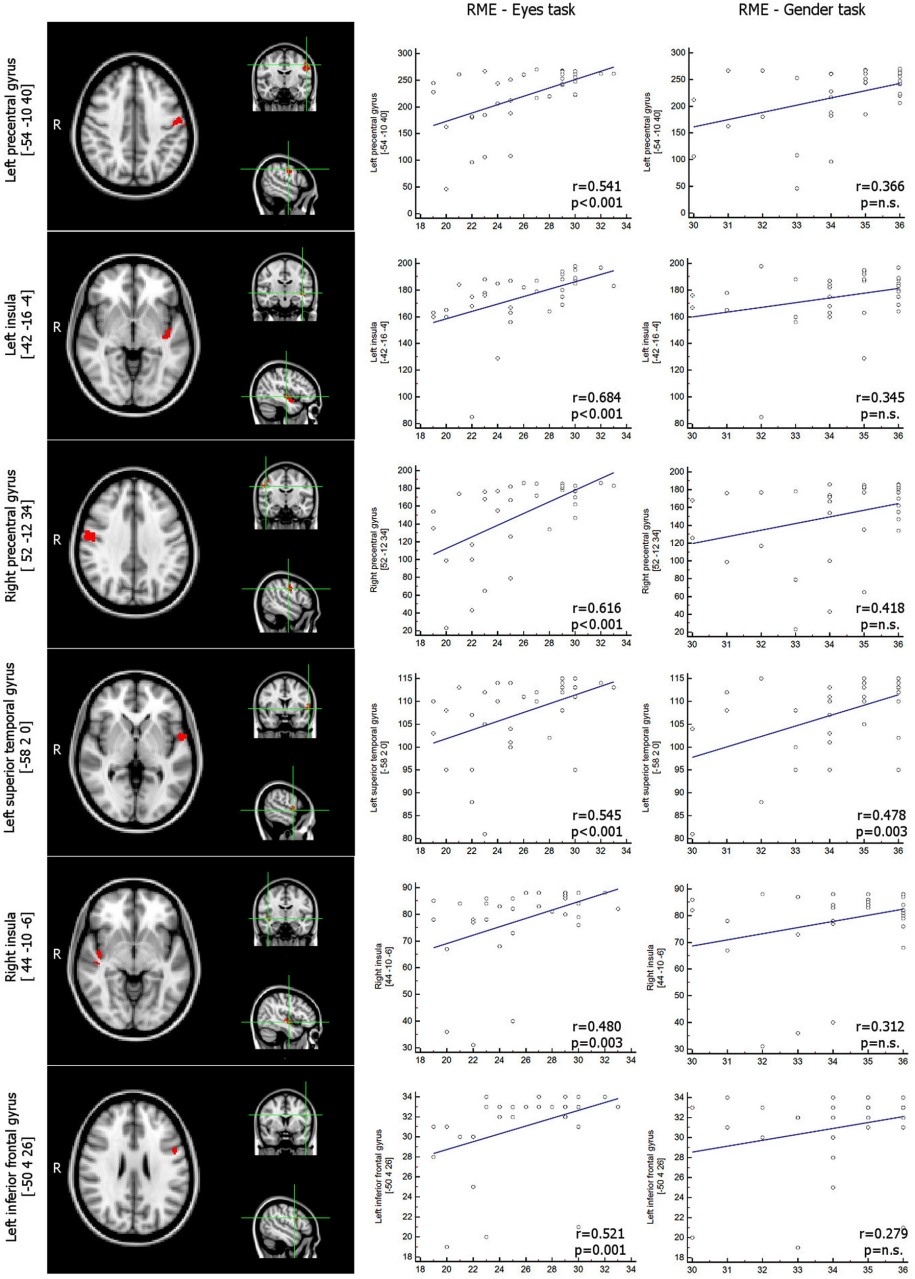
**VBM results.** GM areas inversely related with RME test, *p* < 0.05_corrected_ (among those areas that were inversely related with age, thresholded at *p* < 0.05_corrected_) – Scatterplot representing the correlation between number of voxels within each significant GM cluster and scores in RME-Eyes task and in RME-Gender task. Coordinates are in MNI space. VBM, Voxel-based Morphometry; GM, Gray Matter; RME, Reading the Mind in the Eyes test.

The correlation analysis between GM areas (masked with results of correlation with age) and results in the RME test showed a direct correlation in bilateral precentral gyri, the bilateral posterior insula, the left inferior frontal and left superior temporal gyri (**Figure 2**; **Table 2**). No areas were found to be inversely correlated with RME test.

**Table 2:** Voxel-based morphometry clusters of direct correlation between gray matter volume and RME test (*p* < 0.05_corrected_ threshold), among the cortical areas whose volume decreases with age.

Cluster size	*P*_max_	Peak cluster MNI coordinates (*x, y, z*)			Center of gravity MNI coordinates (*x, y, z*)			Brain area
271	0.002	-54	-10	40	-54.7	-9.06	34.4	Left precentral gyrus - BA 4
210	0.007	-42	-16	-4	-40.4	-7.37	-10.9	Left insula - planum polare
186	0.002	52	-12	34	54	-8.03	37.2	Right precentral gyrus - supramarginal gyrus
115	0.004	-58	2	0	-56.3	1.48	3.46	Left superior temporal gyrus, BA 22
90	0.009	44	-10	-6	44.1	-13.4	-3.84	Right insula - planum polare
34	0.029	-50	4	26	-51.4	9.05	23.2	Left inferior frontal gyrus BA 44/BA 6


As expected, the statistical correlation between ICI and RME test showed a direct correlation in each of the considered clusters, whereas the correlation between ICI and RME gender test was statistically significant only in the left superior temporal gyrus (*p* < 0.003, rho 0,478; **Figure 1**; **Table 3**).

**Table 3:** Correlation analyses between ICI data computed on GM areas whose volume correlated with RME test score (among those GM areas whose volume negatively correlated with age) and RME test, RME gender test and LFT.

	RME test		GENDER test		LFT	
			
	Correlation coefficient	*p*-value	Correlation coefficient	*p*-value	Correlation coefficient	*p*-value
Left precentral gyrus [ICI]	0.541	0.0007	0.366	n.s.	0.113	n.s.
Left insula [ICI]	0.684	<0.0001	0.345	n.s.	-0.106	n.s.
Right precentral gyrus [ICI]	0.616	0.0001	0.418	n.s.	-0.259	n.s.
Left superior temporal gyrus [ICI]	0.545	0.0006	0.478	0.003	0.116	n.s.
Right insula [ICI]	0.480	0.003	0.312	n.s.	-0.166	n.s.
Left inferior frontal gyrus [ICI]	0.521	0.001	0.279	n.s.	-0.246	n.s.


*RME, Reading the Mind in the Eyes test; ICI, Individual Cluster Index; LFT, Letter Fluency Task; GM, Gray Matter.*

The correlation between LFT test and ICI in the subgroup of elderly subjects was not significant in any of the considered clusters.

### White Matter Analysis – TBSS

Tract-based spatial statistics analyses showed an inverse correlation between age and FA-values, involving most of the brain WM areas. In contrast, we did not find areas of WM whose FA-values were directly correlated with the same variable.

**FIGURE 3:**
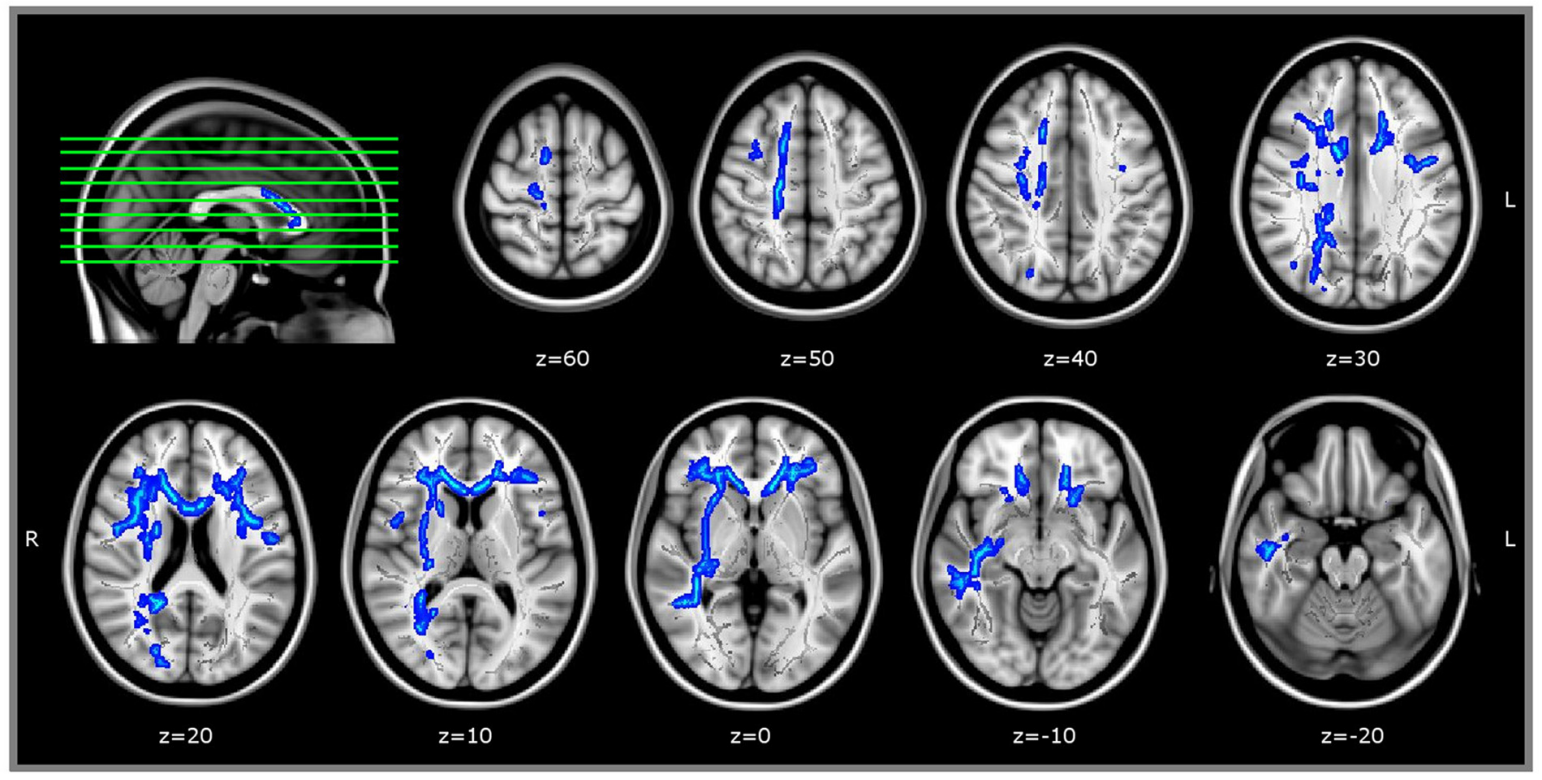
**TBSS results.** WM areas whose FA-value was directy correlated with RME test, *p* < 0.05_corrected_ (within areas inversely related with age, thresholded at *p* < 0.05_corrected_). Coordinates are in MNI space. TBSS, Tract-Based Spatial Statistics; FA, Fractional anisotropy; RME, Reading the Mind in the Eyes test.

Regression analysis between WM microstructure (FA-value) and RME test score (within WM areas that correlated with age) revealed a direct correlation between FA and RME test in bilateral frontal areas anatomically compatible with the right superior longitudinal fasciculus and, only in right hemisphere, with the fronto-temporal parts of superior longitudinal fasciculus. We found a direct correlation with FA-values also in bilateral uncinate fasciculus, right inferior fronto-occipital fasciculus, right inferior longitudinal fasciculus, and with the genu of the corpus callosum (JHU White-matter tractography Atlas, FSL; **Figure 3**). We did not find any WM area whose FA-value was inversely correlated with RME score.

## Discussion

The eye gaze offers important information for decoding the mental and affective states of a person. In this MRI study we examined how brain structural changes that correlate with aging affect the Mind-Reading ability through the eyes within a sample of subjects from 24 to 79 years of age.

Behavioral results showed that all participants performed within the normal range on the RME test, suggesting that the Mind-Reading ability is preserved with age, as also demonstrated by previous research (Phillips et al., 2002; Castelli et al., 2010). However, investigating the relationship between age and RME score, we found a significant inverse correlation. This finding is consistent with other evidences reporting that elderly people perform worse than young people on the RME test (Slessor et al., 2007) although this decline is not as important as the one observed in cognitive ToM tasks (McKinnon and Moscovitch, 2007; Slessor et al., 2007; Pardini and Nichelli, 2009; Castelli et al., 2010). This is probably due to the minimal involvement of general cognitive abilities and executive functions in the RME test (Sandoz et al., 2014). Finally, executive functions performance did not correlate with brain areas changing with age and with the RME test performance, thus suggesting that the Mind-Reading performance is independent from general cognitive functioning.

The novelty of our study resides in the investigation of the relationship between areas of GM/WM that change with age and the performance at the RME test. In keeping with literature data, we found age-related cortical and subcortical modifications mainly involving fronto-temporal regions (Raz et al., 1998; Resnick et al., 2000, 2003; Sullivan and Pfefferbaum, 2006). Within these regions we found a direct correlation with RME performance in the bilateral precentral gyri, the bilateral posterior insula, the left inferior frontal and left superior temporal gyri. Moreover, WM results showed a direct correlation between FA and the RME test in clusters localized in frontal and temporal lobes that can be considered as part of the superior longitudinal fasciculus, the inferior fronto-occipital fasciculus and the inferior longitudinal fasciculus, all of them being considered as WM bundles connecting the frontal lobe with temporal and occipital lobes (Mori et al., 2005). Finally, we also found a WM cluster within the anterior corpus callosum, important for the connectivity of the frontal lobes (Mori et al., 2005).

Given the anatomical relationship between the WM and the GM above-mentioned clusters, it is possible that the reduction in RME test performance (although remaining within the range of normality) is driven by a more general loss of connectivity between inferior frontal regions and insular cortices reasonably mediated by the loss in microstructural integrity (as assessed by TBSS) in the fronto-temporal portions of the superior longitudinal fasciculus. These results can be related to the “anterior connectivity cluster” of the TPJ, which connects the inferior frontal gyrus, the anterior insula and the supplementary motor area with TPJ within a network that has been demonstrated to be fundamental for social abilities and to be related to the perceptual and pre-reflective aspects of Mind-Reading (Mars et al., 2012; Herbet et al., 2014, 2015).

Going into more detail about the GM results, our findings showed that the cortical volume of bilateral insula is correlated with both age (inversely) and performance on the RME test (directly). This finding is in line with evidences that associate this area with emotion recognition (Damasio et al., 2000; Phan et al., 2002; Britton et al., 2006; Kurth et al., 2010; Dal Monte et al., 2013), RME test performance (Boucher et al., 2015) and with the difficulty observed in aged subjects in decoding facial expression of emotions (MacPherson et al., 2006; Chaby and Narme, 2009).

Noteworthy, our results also show an involvement of the precentral gyrus, particularly the premotor cortex among those areas that change with age and are directly correlated with RME test. This area is involved in action planning and is considered a core region of the MNS (Rizzolatti et al., 2001). The importance of the MNS within ToM ability was stated by the interactive dual-process theory (Coricelli, 2005; Keysers and Gazzola, 2007; Uddin et al., 2007), that postulates that mentalizing abilities emerge from the interaction between two distinct neural networks: the MNS, involved in the low-level embodied representation and pre-reflexive perceptual processes (i.e., emotional empathy, basic emotion recognition, and motor intention decoding) and the mentalizing network, which is related to an higher level representation involved in the attribution of mental states to other people. A great number of studies found evidences that the MNS informs and supports the Mind-Reading ability (Decety and Chaminade, 2003; Blakemore et al., 2004; Agnew et al., 2007; Keysers and Gazzola, 2007; Castelli et al., 2010) and this interaction is hypothesized to be driven by the premotor cortex, which identifies goals and intentions of others (Rizzolatti et al., 2002). The connectivity between MNS and mentalizing network is mediated by perisylvian components of the superior longitudinal fasciculus: the arcuate fasciculus and the lateral-superficial part of superior longitudinal fasciculus (Catani and Jones, 2005; Makris et al., 2005; Rilling et al., 2008; Martino et al., 2013) whose FA we found to correlate with age and performance in RME test. Both the arcuate and the lateral superior longitudinal fasciculi are important for social cognition (Barbey et al., 2012). Finally, a recent lesion study found that the behavioral performance at the RME test is inversely correlated with the degree of disconnection of the right perisylvian network (Herbet et al., 2014, 2015).

Our VBM results also show the involvement of the inferior frontal gyrus among the age-related areas whose volume change with RME test performance. This area has been recently demonstrated to be necessary for the RME performance (Dal Monte et al., 2014), for tuning into others’ affective state of mind (Chakrabarti et al., 2006) and for language production (Price, 2010). Moreover, we found that cortical volume of the superior temporal gyrus together with the FA values of WM bundles connecting this area with the inferior frontal gyrus were associated with both age and RME performance. These results can be explained by the verbal components of the RME test that requires explicit labeling of the eyes expressions depicted in the pictures. Indeed, it has been demonstrated that Mind-Reading ability, although it implies largely implicit mentalizing processes, is influenced in part by other cognitive abilities, first of all language skills. Peterson and Miller (2012) showed that in a healthy adult sample with a mean intelligence quotient (IQ) score, the verbal IQ (which measures basic vocabulary knowledge as well as other expressive verbal skills) alone accounted for almost 25% of the variance in the RME test performance (Peterson and Miller, 2012).

Interestingly, despite the observation of a cortical volume reduction in language-specific areas such as inferior frontal gyrus and superior temporal gyrus and decreased FA in the underlying connecting WM bundles, the Mind-Reading ability is relatively well preserved with increasing age. This result is in keeping with previous research showing that language abilities, especially language comprehension, are among the ones best preserved in elderly people (Burke and Shafto, 2004; Tyler and Marslen-Wilson, 2008) and do not decline proportionally to the amount of atrophy in fronto-temporal regions (Park et al., 2002). This is mediated by the involvement of bilateral language-area that compensate this volume reduction (Cabeza, 2002; Reuter-Lorenz, 2002) and allow older people to complete the RME task successfully.

## Conclusion

The aging-related decline in the Mind-Reading ability through the eyes could be a direct consequence of brain structural and functional changes, such as GM tissue loss and microstructural changes in WM areas, as discussed above.

Given this premise it is possible to speculate that, in healthy aging, volume reduction at the level of premotor cortex, inferior frontal gyrus, insula and superior temporal gyrus, associated with a decrease of the connections between frontal and temporal cortices, might result in a lower ability to understand the others mental states in a rapidly and intuitively way, as the Mind-Reading ability allows us to do. However, the recruitment of additional neural areas and circuits, such as bilateral language areas, might help preserve Mind-Reading performance. Our results are coherent with a previous fMRI study by our group (Castelli et al., 2010) showing that there were no differences in the RME test performance between young and old subjects, but that the elderly had increased activation, compared to young subjects, in the same network that we found in this work: the premotor cortex, the inferior frontal gyrus and the temporo-insular regions. To conclude, structural brain changes in GM and WM may explain the differences in the Mind-Reading ability across our sample and that adapted neural plasticity such as compensatory mechanisms might overcome the age-related cortical loss and account for the intact Mind-Reading through the eyes performance of elderly people (Cabeza, 2002; Park and Reuter-Lorenz, 2009; Castelli et al., 2010; Cramer et al., 2011).

## Conflict of Interest Statement

The authors declare that the research was conducted in the absence of any commercial or financial relationships that could be construed as a potential conflict of interest.
